# *In vitro* expression of *Streptococcus pneumoniae ply* gene in human monocytes and pneumocytes

**DOI:** 10.1186/s40001-015-0142-4

**Published:** 2015-05-07

**Authors:** Da-Kang Hu, Yang Liu, Xiang-Yang Li, Ying Qu

**Affiliations:** Department of Laboratory Medicine, Taizhou Municipal Hospital, 381# Zhongshan East Road, Taizhou, 318000 China; Department of Laboratory Medicine, The First Affiliated Hospital of Nanchang University, 17# Yong Wai Zheng Street, Nanchang, 330006 China; Department of Laboratory Medicine, The Second Affiliated Hospital of Wenzhou Medical University, 109# College West Road, Wenzhou, 325027 China

**Keywords:** Streptococcus pneumoniae, Infection, Virulence gene, Pneumolysin (*ply*)

## Abstract

**Background:**

*Streptococcus pneumoniae* is one major cause of pneumonia in human and contains various virulence factors that contribute to pathogenesis of pneumococcal disease. This study investigated the role of pneumolysin, Ply, in facilitating *S. pneumoniae* invasion into the host blood stream.

**Methods:**

*S. pneumoniae* strains were isolated from clinical blood and sputum samples and confirmed by PCR. Expression of *ply* gene was assessed by infecting human monocytes and pneumocytes.

**Results:**

A total of 23 strains of *S. pneumoniae* isolated from blood (*n* = 11) and sputum (*n* = 12) along with *S. pneumoniae* ATCC49619 were used to infect human monocyte (THP-1) and Type II pneumocyte (A549) cell lines. All clinical strains of *S. pneumoniae* showed higher expression of *ply* mRNA than the American Type Culture Collection (ATCC) strain. Among the clinical strains, blood isolates showed higher expression of *ply* genes than sputum isolates, i.e., 2^1.5^- to 2^1.6^-folds in THP-1 and 2^0.4^- to 2^4.9^-folds in A549 cell lines.

**Conclusions:**

The data from the current study demonstrated that *ply* gene of blood- and sputum-derived *S. pneumoniae* is differentially expressed in two different cell lines. Under survival pressure, *ply* is highly expressed in these two cell lines for blood-derived *S. pneumoniae*, indicating that *ply* gene may facilitate *S. pneumoniae* invasion into the host blood system.

## Background

*Streptococcus pneumoniae* (*S. pneumoniae*) is a gram-positive diplococcus that is the main pathogenic bacterium of community-acquired pneumonia, otitis media, meningitis, abscesses, and other infections, particularly in infants and the elderly [1]. In developing countries, up to 1 million deaths annually are caused by pneumonia in children less than 5 years of age, in which *S. pneumoniae* is the major pathogenic bacterium [2]. Normally, *S. pneumoniae* is colonized in the respiratory tract of asymptomatic carriers as an opportunistic bacterium [3]. And it is difficult to predict when it converts to pathogenic bacterium although Manso *et al.* [4] reported that such a phase variation consists of genetic rearrangements. However, when the host immunosystem becomes weak, *S. pneumoniae* will be infectious. During *S. pneumoniae* conversion from an opportunistic bacterium to a particularly pathogenic bacterium that causes severe invasive infections such as blood and cerebrospinal fluid (CSF) infections, the complex interaction will occur between *S. pneumoniae* and the body’s immune system. In addition, infection could also be due to acquisition of virulent serotypes not previously part of the colonizing serotypes, which is named as transformation. Multiple components of *S. pneumoniae*, such as capsule and other virulence factors are involved in triggering the host immune responses [5-7], while a variety of cells in the host immune system (such as neutrophils, monocytes/macrophages, and dendritic cells) will be activated to defend against the infection by either killing the bacteria or releasing a variety of factors (such as IL-1α, IL-1β, IL-6, IFN-α, IL-8, and ICAM-1).

The septicemia and meningitis caused by *S. pneumoniae* are much more fatal than that of other types of infections [8]. However, few studies have been reported that focus on the mechanism responsible for *S. pneumoniae* invasion into the host blood system and nervous system [7]. Mahdi *et al.* [9] confirmed the role of transcription factor SP_0927 in pathogenesis and virulence. Uchiyama *et al.* [7] reported that neuraminidase A (NanA) is a virulence factor that facilitates *S. pneumoniae* invasion into the CSF. Ricci *et al.* [10] reported that pneumococcal surface protein A (PspA) contributes to pneumococcal meningitis rather than pneumococcal surface protein C (PspC). Based on previous studies on *S. pneumoniae* infection, different virulence factors in *S. pneumoniae* are able to induce multiple reactions from the host immune system. To date, there are more than 10 virulence factors discovered in *S. pneumoniae*, including NanA, capsular polysaccharide synthesis A (CpsA), choline-binding protein A (CbpA), pneumococcal surface adhesion A (PsaA), PspA, PspC, pneumolysin (Ply), and so on [2]. Different virulence factors may have different functions in contributing to the different types of *S. pneumoniae* infections.

Furthermore, study on *S. pneumoniae* virulence factors uses human cell lines, such as monocytes or lung epithelial cells. These types of cells are important in our body to prevent or defend against *S. pneumoniae* infection. The major role of monocytes is to kill bacteria and to trigger leukocyte chemotaxis for immunoreactions, while lung epithelial cells may function as an antigen presenter. In addition, type II pneumocytes have long been known to synthesize and secrete complement component C3, providing a target for *S. pneumoniae* adherence to those cells *via* CbpA. Thus, both types of cells are the most commonly used for study of the pathogenic mechanism of *S. pneumoniae in vitro*. To date, there is no report showing which specific virulence factor facilitates *S. pneumoniae* invasion into the host blood system. Possibly, different virulence factors play different roles in disease progression. In this paper, only *ply* expression is shown. We first isolated *S. pneumoniae* (23 strains) from clinical blood and sputum samples. They then infected monocytes or lung epithelial cell lines and the levels of *ply* mRNA expression were analyzed.

## Methods

The experimental protocol was established according to the ethical guidelines of the Helsinki Declaration and was approved by the Human Ethics Committee of Taizhou Municipal Hospital, China. Written informed consent was obtained from individual participants.

### Isolation of *S. pneumoniae* strains

The standard *S. pneumoniae* strain ATCC49619 was provided by the Chinese National Center for Medical Culture Collections (Beijing, China). Twenty-three clinical *S. pneumoniae* strains were isolated from different in-patients at the Second Affiliated Hospital of Wenzhou Medical University (Wenzhou, Zhejiang, China) in 2009, which were preserved in 20% glycerin Luria-Bertani Medium and stored at −80°C; among them, 11 strains were isolated from blood samples, called blood-derived *S. pneumoniae*, and the other 12 strains were isolated from sputum samples, called sputum-derived *S. pneumoniae*. All 23 strains had no duplication and only one strain was isolated from each selected patient. All *S. pneumoniae* strains were identified by the Gram-Positive Identification Card (GPI) of VITEK-32 automatic microorganism analyzer (bioMérieux Co., Marcy-Etoile, France) and confirmed by PCR according to du Plessis [11]*.*

### *S. pneumoniae* culture

A total of 24 *S. pneumoniae* strains were cultured on blood agar plates at 37°C in a 5% CO_2_ incubator (Thermo Electron Co., Waltham, MA, USA) and adjusted to 1.0 McFarland in normal saline, which is used for infection experiments and RNA extraction.

### Cell line and culture

Human monocytes THP-1 and type II pneumocytes A549 cell lines were obtained from the Cell Bank of Chinese Academy of Sciences (Shanghai, China) and cultured with RPMI 1640 and F-12 K medium (GIBCO, CA, USA), respectively, and 10% fetal bovine serum (Zhejiang Tianhang Biological Technology Co., Zhejiang, China) plus 1% strep-penicillin (Beijing Solarbio Science & Technology Co., Beijing, China) in a 37°C humidified incubator (Thermo Electron Co., Waltham, MA, USA) with 95% air and 5% CO_2_. THP-1 and A549 cells were prepared at concentrations of 4.0 and 3.0 × 10^8^/L respectively for the infection experiments and such preparation was conducted between May 2011 and July 2011 at the Center Laboratory of Experiments, Wenzhou Medical University.

### Infection of THP-1 and A549 cell lines with *S. pneumoniae* cultures

One milliliter of THP-1 cells was grown in 24-well culture plates, which were 4.0 × 10^8^/L. One milliliter of *S. pneumoniae* cultures from clinical samples and American Type Culture Collection (ATCC) strains was then seeded into the 24-well culture plates, which was 1.0 McFarland. The blank control used normal saline instead of *S. pneumoniae*. The cells were maintained at 37°C in 5% CO_2_ for 4 h (25 wells) and 8 h (other 25 wells). At the end of the experiments, all of the cell cultures plus controls were transferred into 1.5-ml centrifuge tubes and centrifuged for 10 min at 4,000 rpm and then subjected to RNA isolation and PCR analysis. For A549 cell cultures, 50 μl of 0.25% trypsin (GIBCO, Pleasanton, CA, USA) were used to digest the adherent cells after the suspensions were harvested, whereas there was no trypsin used for THP-1 cell suspension culture. The experiments were repeated at least once and all the 24 *S. pneumoniae* strains were studied in these two cell lines.

### Semi-quantitative RT-PCR

Total RNA from *S. pneumoniae* culture was isolated using a RNA extraction kit from Takara (Dalian, China) according to the manufacturer’s protocols. The purity of RNA was determined by the Nanodrop 2000 spectrophotometer (Thermo Electron Co., Waltham, MA, USA) and the optical density ratios of OD260/OD280 were all between 1.8 and 2.2. After that, these RNA samples were converted to cDNA using 2.0 μl of 5x PrimeScript Buffer, 0.5 μl PrimeScript Buffer RT Enzyme Mix I, 0.5 μl Oligo dT Primer (50 μM), 0.5 μl Random 6 mers (100 μM), 5.0 μl total RNA, and 1.5 μl RNase free ddH_2_O for 15 min at 37°C and then stopped at 85°C for 5 s. After that, real-time PCR analysis was performed by using 10.0 μl SYBR Premix Ex Taq II (Takara), 0.8 μl PCR primer each (10 μM), 0.4 μl ROX Reference Dye II (50x), 2.0 μl RT mixture, and 6.0 μl ddH_2_O for pre-degeneration at 95°C for 30 s and then 40 cycles of 95°C for 5 s and 60°C for 34 s in a 7,500 fluorescence quantitative PCR instrument (ABI, San Ramon, CA, USA). The *ply* primers were synthesized according to a previous study [12] (5′-GATGGCAAATAAAGCAGTAAATGACT-3′ and 5′-TGATGCCACTTAGCCAACAAATCG-3′). The 16S rRNA primers were synthesized according to reference [13]: (5′-GGTGAGTAACGCGTAGGTAA-3′ and 5′-ACGATCCGAAAACCTTCTTC-3′). All the primers were synthesized at Shinegene Co. (Shanghai Shanjing, China). Levels of 16S rRNA were used as an internal reference to quantify the level of *ply* expression in this study. The blank control was from non-infected cell lines to replace *S. pneumoniae* with normal saline. PCR amplification data were quantified by using ABI 7500 Software v2.0.1. while ‘Threshold’ and ‘Baseline’ were selected as ‘auto’.

### Statistical analysis

SPSS17.0 statistical software (SPSS, Chicago, IL, USA) was used for all statistical analyses. The normality analysis was performed by using the Kolmogorov-Smirnov test, and the homogeneity of variance analysis between two groups using Levene’s test. The mean difference between clinical *S. pneumoniae* (including blood and sputum *S. pneumoniae* samples) and control or ATCC49619 was analyzed using the single-sample *t* test, and the comparison of blood- and sputum-derived *S. pneumoniae* samples using analysis of variance for factorial designs in infection of A549 cells. In addition, the two-sample *t* test was used to determine whether there is a difference in virulence factor expressions between blood group and sputum group of *S. pneumoniae* before the infection. The Cochran & Cox separate variance estimation *t* test was used among blood or sputum groups of *S. pneumoniae* after the infection of THP-1 cells. *P* > 0.10 in the normality test, *P* > 0.10 in the variance homogeneity test, and *P* < 0.05 in mean comparison were considered statistically significant.

## Results

### Expression of virulence gene *ply* in *S. pneumoniae* after infecting THP-1 and A549 cells

To detect the changes in expression of virulence gene *ply* in these *S. pneumoniae* samples, we used the *S. pneumoniae* samples to infect THP-1 and A549 cells for 4 h and 8 h, respectively.

As shown in Table 1, ∆Ct values of the virulence gene *ply* were significantly different after blood-derived *S. pneumoniae* infected THP-1 cells for 4 h and 8 h compared to pre-infection, but there was no significant difference between 4 h and 8 h infection. However, there was no statistically significant difference in expression of *ply* after sputum-derived *S. pneumoniae* infected THP-1 cells for 4 h and 8 h compared to pre-infection, but the expression was significantly higher after 8 h infection. However, compared to the ATCC49619 control, before and after blood- or sputum-derived *S. pneumoniae* infected THP-1 cells, ∆Ct values of virulence gene *ply* had a significant difference, indicating that the expression of *ply* was weaker than ATCC49619 before infection, but expression of *ply* was higher than ATCC49619 after infection. Between blood- and sputum-derived *S. pneumoniae*, ∆Ct of *ply* expression were significantly different after they infected THP-1 cells for 8 h: after infection in THP-1 cells for 8 h, *ply* expression of blood-derived *S. pneumoniae* was stronger than that of sputum-derived ones. Similar results of *ply* expression were obtained from blood-derived or sputum-derived *S. pneumoniae* infecting A549 cells for 4 h and 8 h (Table 1).

**Table 1:** **Expression of virulence gene** ***ply*** **after** ***S. pneumoniae*** **infected THP-1 or A549 cells**

**Groups**	***n***	***ply*** **(△Ct,‾x ±** ***s*** **)**				
		**0 h**	**4 h (THP-1)**	**8 h (THP-1)**	**4 h (A549)**	**8 h (A549)**
ATCC	1	16.2	31.5	27.9	21.8	19.1
Blood SP	11	29.2 ± 2.6	24.8 ± 3.2	23.0 ± 1.5	20.4 ± 5.1	18.2 ± 2.9
Sputum SP	12	28.1 ± 2.9	26.3 ± 2.7	24.6 ± 1.4	20.8 ± 3.0	23.3 ± 3.1

Note: △Ct = Mean Ct of tested gene − Mean Ct of control gene. Smaller △Ct means more *ply* expression. Level of 16S rRNA was used as a control, which expresses much higher than that of *ply*. SP, *Streptococcus pneumoniae*; ATCC, American Type Culture Collection*.*

In A549 cells, there was no statistically significant difference in expression of virulence gene *ply* between the following groups: the blood group *vs*. the ATCC49619 standard in 4 h or 8 h cultures, the sputum group *vs*. the ATCC49619 standard in 4 h cultures (*t* = −0.901, −1.049, −1.206, respectively; *P* = 0.389, 0.319, 0.253, respectively). In contrast, the remaining comparisons showed significant differences (*P* < 0.05) (Tables 1 and 2). Moreover, after both blood- and sputum-derived *S. pneumoniae* infected A549 cells, there were statistically significant differences in expression of virulence gene *ply* between blood- and sputum-derived *S. pneumoniae* (*F* = 6.560, *P* = 0.014), but 4 h to 8 h culture did not show a significant difference (*F* = 0.025, *P* = 0.874), analyzed by a variance of factorial designs. However, interaction between samples (including blood and sputum samples) and infection time points had statistical significance (*F* = 5.061, *P* = 0.030).

**Table 2:** ***P*** **values between the following compared groups after** ***S. pneumoniae*** **infected THP-1 cells**

**Compared groups**	***t***	***P*** **value**
0 h B-SP (*n* = 11) *vs*. 0 h S-SP (*n* = 12)	0.956	0.350
4 h B-SP (*n* = 11) *vs*. 4 h S-SP (*n* = 12)	−1.223	0.235
8 h B-SP (*n* = 11) *vs*. 8 h S-SP (*n* = 12)	−2.690	0.014
0 h B-SP (*n* = 11) *vs*. 4 h B-SP (*n* = 11)	2.778	0.020
0 h B-SP (*n* = 11) *vs*. 8 h B-SP (*n* = 11)	6.775	<0.001
4 h B-SP (*n* = 11) *vs*. 8 h B-SP (*n* = 11)	1.578	>0.10
0 h S-SP (*n* = 12) *vs*. 4 h S-SP (*n* = 12)	1.561	0.147
0 h S-SP (*n* = 12) *vs*. 8 h S-SP (*n* = 12)	4.341	0.001
4 h S-SP (*n* = 12) *vs*. 8 h S-SP (*n* = 12)	2.034	>0.05
0 h B-SP (*n* = 11) *vs*. 0 h ATCC (*n* = 1)	16.449	<0.001
4 h B-SP (*n* = 11) *vs*. 4 h ATCC (*n* = 1)	−6.908	<0.001
8 h B-SP (*n* = 11) *vs*. 8 h ATCC (*n* = 1)	−11.154	<0.001
0 h S-SP (*n* = 12) *vs*. 0 h ATCC (*n* = 1)	14.350	<0.001
4 h S-SP (*n* = 12) *vs*. 4 h ATCC (*n* = 1)	−6.706	<0.001
8 h S-SP (*n* = 12) *vs*. 8 h ATCC (*n* = 1)	−7.859	<0.001

Note: SP, *Streptococcus pneumoniae;* B-SP, blood SP; S-SP, sputum SP; ATCC, American Type Culture Collection.

Table 2 showed *P* values between the following compared groups after *S. pneumoniae* infected THP-1 cells due to non-homogeneity of variance. Cochran & Cox separate variance estimation *t* test was used to compare blood and sputum groups of *S. pneumoniae* after the infection.

### Changes in THP-1 and A549 cell morphology after *S. pneumoniae* infection

**Figure 1:**
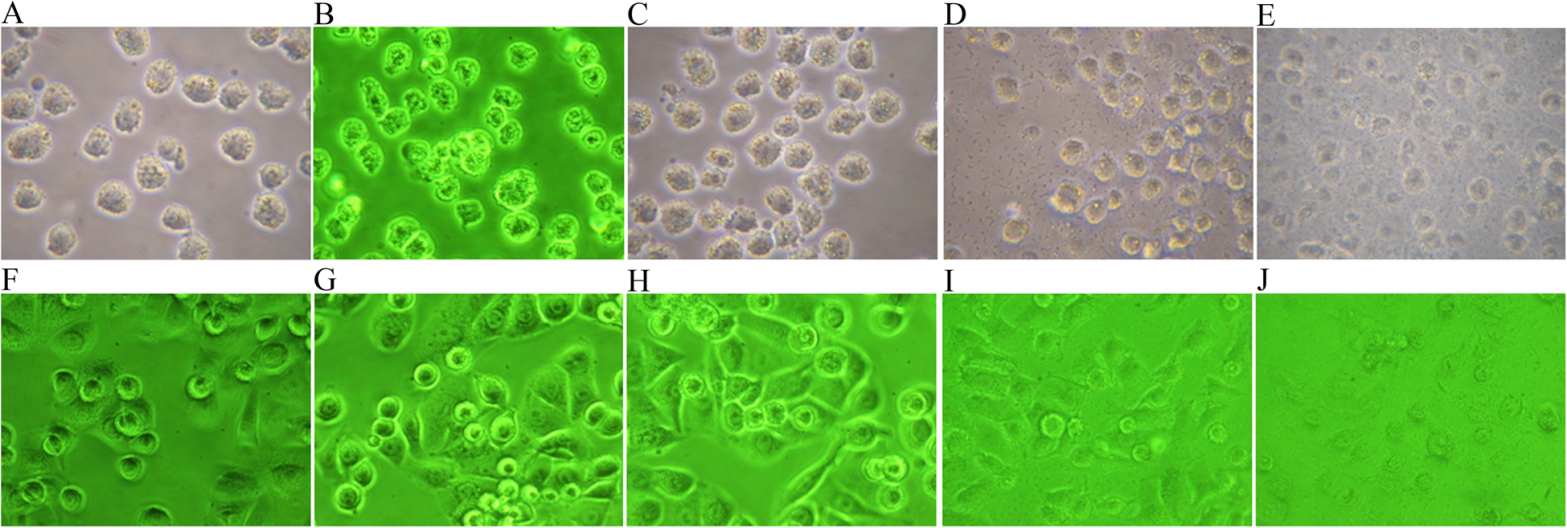
Morphological changes of THP-1 and A549 cells after *Streptococcus pneumoniae* (SP) infection. THP-1 and A549 cells began to die after infection at 4 to 8 h: **(A)** THP-1 at 0 h; **(B)** THP-1 (uninfected control) at 4 h; **(C)** THP-1 (uninfected control) at 8 h; **(D)** THP-1 with SP at 4 h; **(E)** THP-1 with SP at 8 h; **(F)** A549 at 0 h; **(G)** A549 (uninfected control) at 4 h; **(H)** A549 (uninfected control) at 8 h; **(I)** A549 with SP at 4 h; **(J)** A549 with SP at 8 h. (A-J: ×400). One strain of blood-derived SP was used except in the uninfected control. 0 h means before infection.

As shown in Figure 1, the longer time of THP-1 cells culture with *S. pneumoniae*, the more deaths THP-1 cells suffered, levels of which were associated with different sources of *S. pneumoniae* isolations and amounts. Similar data were observed in A549 cell cultures of these *S. pneumoniae* samples.

## Discussion

In the current study, we first isolated and confirmed 23 *S. pneumoniae* strains from clinical blood and sputum samples. We then used these 23 *S. pneumoniae* strains and ATCC49619 to infect human monocytes and pneumocytes and then analyzed expression of the *S. pneumoniae* virulence gene *ply* in these 23 strains and ATCC49619. We found that levels of *ply* mRNA expression were much higher in clinical *S. pneumoniae* samples than in the ATCC49619 standard and in blood-derived *S. pneumoniae* samples than in sputum-derived ones. This finding demonstrated differential expression of *ply* mRNA levels in blood-derived *S. pneumoniae* after infecting THP-1 and A549 cells compared to sputum-derived ones. This study provides indirect molecular evidence that the *S. pneumoniae* virulence gene *ply* may facilitate *S. pneumoniae* invasion into the host blood system.

Indeed, Ply is a pore-forming toxin with a molecular weight of 53 kD and is known as a cholesterol-binding cytolysin, a key virulence factor for clinical *S. pneumoniae* strains. Ply protein can dissolve cell membranes in almost all eukaryotes. Specifically, Ply toxin will bind to a cholesterol-rich membrane using its pore-forming mechanism and then penetrate into the lipid bilayer of the cell membrane and oligomerize to form a perforation on the cell membrane [14]. Ply can also activate macrophages to execute apoptosis without membrane pore formation [15]. Thus, Ply toxin has a wide range of biological activities, such as facilitation of *S. pneumoniae* colonization and pathogenicity. Ply can also activate the classic complement pathway in the human body, inhibit cough and bactericidal activity and migration of white blood cells [16], stimulate IL-8 synthesis [17], and increase ICAM-1 and IL-1β secretions [18-20]. The Ply impact on the immune response to the pneumococcus is highly dependent on the strain background, thus, it is surely important of the interaction between specific virulence factors and other components of the genetic background of this organism [19]. A recent study also confirmed the role of *ply* in biofilm formation, which is separate from the hemolytic activity responsible for tissue damage during pneumococcal disease [21]. Our current data showed that after infecting THP-1 cells, expression of *ply* mRNA was significantly induced in blood-derived *S. pneumoniae* strains compared to sputum-derived ones, indicating that the increased *ply* expression may contribute to *S. pneumoniae* against defense of THP-1 cells, which may be one of the mechanisms for *S. pneumoniae* invasion into the host blood system. In the current study, we determined *ply* expression after infecting two types of human cell lines and data are identical, which are consistent with a previous study [22]. Besides, as a whole, expression of *psaA* and *cpsA* was also studied in a previous study [23]. And different patterns of their expression clearly showed different roles of such three virulence genes. The expression of *cpsA* is the basis of the pathogenicity of SP and *ply* expression is more important than that of *psaA* in SP invasion into the blood system. Our current data demonstrated that inflammation induction of different SP sources tends to be consistent [20]. However, the infection types are determined mainly by different sources of SP itself, especially its expression of virulence genes.

In the current study, we utilized a standard strain of *S. pneumoniae* ATCC49619 for comparison. We found that *S. pneumoniae* isolated from both blood and sputum samples were more virulent by expression of high levels of Ply toxin after infecting THP-1 and A549 cells. ATCC49619 is a standard *S. pneumoniae* strain and its physical and chemical properties are relatively stable. The *S. pneumoniae* isolated from clinical samples were much more easily influenced by external environment than ATCC49619. Thus, compared to clinical *S. pneumoniae*, the levels of virulence and viability of ATCC49619 are weaker, all of which may indicate that clinical *S. pneumoniae* strains are more infectious. However, it remains to be determined whether *S. pneumoniae* isolated from different individuals also have different levels of infection potential.

Moreover, we also showed the interaction between *S. pneumoniae* and THP-1 cells or A549 cells. Different sources of *S. pneumoniae* resulted in different outcomes, which could suggest that various strains of *S. pneumoniae* have different abilities to infect the lung, blood, or even the CSF. However, this study is just a proof-of-principle and much more are needed to investigate other virulence factors of *S. pneumoniae* or their combinations in infecting the human body. Indeed, *S. pneumoniae* have many other virulence genes, such as *nanA* [7], which has been demonstrated to facilitate *S. pneumoniae* invasion into the CSF. Moreover, *S. pneumoniae* invasion into the host blood system is an important step and prerequisite for its further invasion into the CSF in most cases. The role of Ply toxin in *S. pneumoniae* invasion into the host blood system and its relationship with NanA are worthy of further investigation. In addition, the significance of this *in vitro* study needs further confirmation by an *in vivo* study.

## Conclusions

The current study demonstrated that *ply* gene of blood- and sputum-derived *S. pneumoniae* is differentially expressed in two different cell lines. Under survival pressure, higher expression of *ply* gene is needed in these two cell lines for blood-derived *S. pneumoniae*, which indicates that *ply* gene may facilitate *S. pneumoniae* invasion into the host blood system.
